# Cranial bone regeneration according to different particle sizes and densities of demineralized dentin matrix in the rabbit model

**DOI:** 10.1186/s40902-016-0073-1

**Published:** 2016-07-05

**Authors:** Jin-Woo Nam, Moon-Young Kim, Se-Jin Han

**Affiliations:** Department of Oral and Maxillofacial Surgery, College of Dentistry, Dankook University, 119 Dandae-ro, Dongnam-gu, Cheonan, Chungnam South Korea

**Keywords:** Demineralized dentin matrix, Bone regeneration, Osteoinduction, Particle size

## Abstract

**Background:**

The objective of this study was to place bone graft materials in cranial defects in a rabbit model and compare their bone regenerating ability according to the size and density of demineralized dentin matrix (DDM).

**Methods:**

We selected nine healthy male rabbits that were raised under the same conditions and that weighed about 3 kg. Two circular defects 8 mm in diameter were created in each side of the cranium. The defects were grafted with DDM using four different particle sizes and densities: 0.1 mL of 0.25- to 1.0-mm particles (group 1); 0.2 mL of 0.25- to 1.0-mm particles (group 2); 0.1 mL of 1.0- to 2.0-mm particles (group 3); and 0.2 mL of 1.0- to 2.0-mm particles (group 4). After 2, 4, and 8 weeks, the rabbits were sacrificed, and bone samples were evaluated by means of histologic, histomorphometric, and quantitative RT-PCR analysis.

**Results:**

In group 1, osteoblast activity and bone formation were greater than in the other three groups on histological examination. In groups 2, 3, and 4, dense connective tissue was seen around original bone even after 8 weeks. Histomorphometric analysis of representative sections in group 1 showed a higher rate of new bone formation, but the difference from the other groups was not statistically significant. RT-PCR analysis indicated a correlation between bone formation and protein (osteonectin and osteopontin) expression.

**Conclusions:**

DDM with a space between particles of 200 μm was effective in bone formation, suggesting that materials with a small particle size could reasonably be used for bone grafting.

## Background

Recently, dental implants have been used to restore defects in patients who are partially or completely edentulous. The proper placement of implants and adequate bone are necessary for long-term stability and sufficient functionality. In the past, dental implantation was possible under only limited circumstances; however, with improvements in the implant itself, as well as in clinical techniques, bone grafting now enables bone to form even when it is deficient, increasing the rate of success.

Bone graft materials can be classified based on their origin, that is, autogenic, allogenic, xenogenic, and alloplastic [1]. Of these, autogenic bone is considered to be the ideal material, although it is limited in terms of its availability, the need for additional surgery, and complications [2–5]. Therefore, allogenic, xenogenic, and alloplastic types of the bone are used more often, even though allogenic and xenogenic bone are associated with such problems as immune reactions and infection, and alloplastic bone provides merely a frame for bone formation [6–10].

The bone component from the extracted teeth is now being used as bone graft material and comprises 55 % minerals and 45 % organic substances [11]. The minerals mainly consist of low-crystalline hydroxyapatite and beta-tricalcium phosphate (β-TCP), and the organic substances are non-collagenous protein, type I collagen, and bone morphogenetic proteins (BMP) [12]. Because this material is derived from autogenic tissue and is therefore biocompatible, there is no risk of immune reaction, rejection, or disease transmission. In addition, osteoconductivity and osteoinductivity can be expected, and autogenic bone can also be formed in a variety of sizes and shapes [13, 14].

Meanwhile, researchers have attempted to improve the success rate of bone grafting. Bone regeneration involves many variables, including size, type, and handling of the bone graft material; the conformation of the bone defect; and whether or not a membrane is applied. In addition, bone graft materials must have a three-dimensional structure that is similar to that found in vivo, must maximize the attachment and functional role of cells, and have the proper strength [15]. Their porous structure acts as a pathway to allow body fluids, blood vessels, and cells to penetrate the material easily. It must also have sufficient surface roughness to permit the osteoblasts to attach easily and proliferate and must provide space for nutrients. In addition, it helps to combine and restore effective factors in order to form satisfactory bony tissue. A large surface area also maximizes contact with body fluids. Wettability, or the degree to which a liquid can spread over a large surface area, provides an optimal environment for the growth of a new bone and the ability to continue to form the new bone over the long term.

Several researchers have reported that the particle size of bone graft materials plays a key role in activating osteoconduction and affects the quality of the new bone [16–19]. Robinson et al. stated that materials with a small particle size enhance bone formation and accelerate bone resorption and remodeling [20]. Rivault et al. reported that particles in autogenic bone, which are 100 μm, help activate osteoblasts [21]. Shapoff et al. reported that the optimum particle size would be 100 to 300 μm [22]. Pallesen et al. stated that particles 0.5 to 2.0 mm in size were preferable to those measuring 10 mm because they allowed more rapid bone remodeling [23]. However, Hall et al. reported that the size was not significant, and Fucini et al. noted that results did not differ significantly when particles 250 to 500 μm or 850 to 1000 μm were used [24, 25]. Using demineralized, freeze-dried bone in a rabbit model, Urist et al. reported that particles 250 to 420 μm in size interrupted chondrogenesis and ossification, whereas those 1000 to 2000 μm in size were more effective [26]. Although all these researchers showed that the amount of bone formed appeared to be influenced by the particle size of bone graft materials, their findings are still controversial.

An adequate size that permits bone formation on the porous structure of bone graft materials is reported to be about 150 μm, which is the size of the gaps in cancellous bone. Demineralized dentin matrix (DDM) is a favorable material for bone formation because it contains osteoconductive material and has dentinal tubules 1.2 to 2.5 μm in diameter [27, 28]. Moreover, both its porous structure and the space between the materials enhance bone formation.

Our objective in this study was to compare the bone-regenerating ability of DDM as bone graft material by placing it in cranial defects of rabbits using different sizes and densities of this material.

## Methods

### Animal models

The experimental animals included nine healthy male rabbits, which weighed around 3 kg and were raised for a specified period of time under the same conditions. After we had ensured that they had no natural abnormalities over 7 days of their adjustment, the animals were approved for the experiment. The animals were raised in an average room temperature of 22 °C, with a 12-h cycle of light and shade and water and solid food freely available. Animal selection and management, surgical protocol, and preparation followed routines were approved by the Institutional Animal Care and Use Committee at Dankook University, Cheonan, Korea.

### Preparation of human DDM

**Fig. 1:**
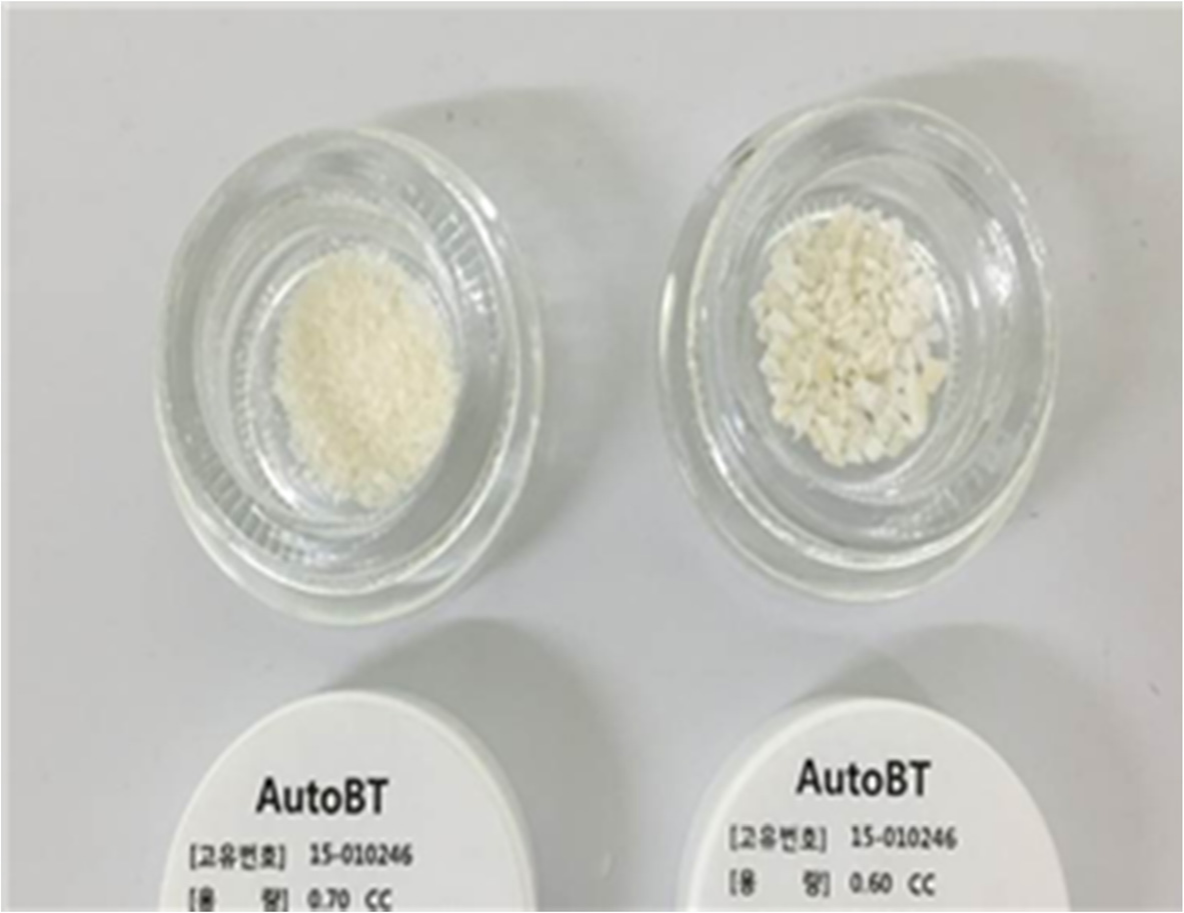
Human demineralized dentin matrix (0.25~1.0 mm, 1.0~2.0 mm)

The teeth extracted from humans were preserved in 70 % ethyl alcohol and shipped to the Korea Tooth Bank (Seoul, Korea), where foreign materials attached to the teeth such as soft tissues or dental calculi were removed. The teeth were then separated into a crown part and a root part, each going through a grinding process to produce particles that were 0.25 to 1.0 mm in size or 1.0 to 2.0 mm in size. These crushed particles were put into a solution of distilled water and hydrogen peroxide, and any foreign materials that remained attached to the particles were removed by means of an ultrasonic cleaner. Cleaned particles were dehydrated with ethyl alcohol and were defatted using an ethyl ether solution. The material was then demineralized for 15 h in hydrochloric acid, washed for 120 min in Tris-hydrochloric acid, and freeze-dried. After these processes had been completed, the particles went through lyophilization and disinfection with ethylene oxide gas and were transported in a covered state to the laboratory and used for transplantation (Fig. 1).

### Surgical procedure

Ketamine HCL (Ketara, Yuhan, Seoul, Korea) 10 mg/kg and xylazine (Rompun, Bayer Korea, Seoul, Korea) 0.15 mg/kg were injected into the rabbit muscle to induce anesthesia, and 2 % lidocaine with 1:100,000 epinephrine was injected at the surgical site for hemostasis; gentamicin sulfate (Dongwha Pharm, Seoul, Korea) 2 mL was injected prior to surgery to prevent infections.

**Fig. 2:**
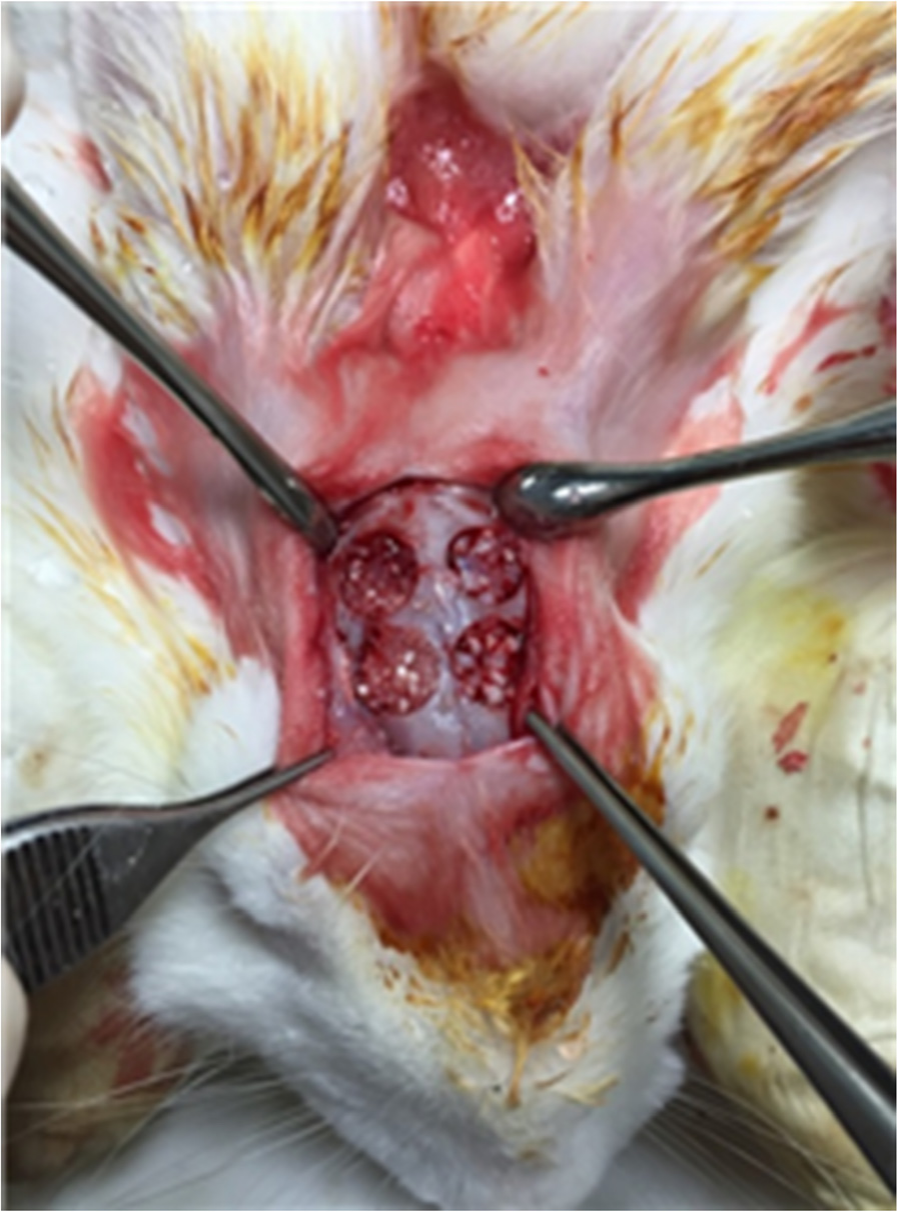
Each group on rabbit calvaria

An incision was made along the midline of the skull to expose the suture area. Periosteum was elevated to the supraorbital region, with care taken not to damage the periosteum. Then, two circular defects, 8 mm in diameter, were created on each side of the skull using a trephine bur, avoiding the nasofrontal suture of the exposed skull so as not to damage the endocranium. A total of four defects were grafted with DDM of different particle sizes and densities (Fig. 2), as follows: 0.1 mL of 0.25- to 1.0-mm particles (group 1); 0.2 mL of 0.25- to 1.0-mm particles (group 2); 0.1 mL of 1.0- to 2.0-mm particles (group 3); and 0.2 mL of 1.0- to 2.0-mm particles (group 4).

The periosteum was sutured with 4-0 Vicryl and the dermis with 3-0 nylon suture. From the day of the operation until day 7, antibiotic ointment was applied on the wounds and gentamicin was injected into the muscles to control infection.

### Production and observation of tissue specimens

The three rabbits were sacrificed after 2, 4, and 8 weeks at each sacrifice. The defects were immediately grafted with DDM, and adjacent tissues were collected and fixed in 10 % buffered formalin for more than 10 days. After the specimens were decalcified using Regular cal solution (6085, BBC Biochemical, Mount Vernon, WA, USA), samples 3 μm in thickness were produced via ethanol dehydration and were embedded in paraffin and stained with hematoxylin-eosin and Masson’s trichrome stains. Digital images were taken using a Panoramic MIDI Scanner (3DHISTECH, Budapest, Hungary).

### Optical microscopic observation

Tissue samples were observed under an optical microscope (Olympus CX41, Olympus, Tokyo, Japan). Proliferation and inflammation of the connective tissues that had formed in space between the bone and graft materials were evaluated, as was evidence of fusion and osteogenesis. The external parts of the bone defects were not examined, because new bone formation in those cranial areas where normal bone tissues are present had little relation to the transplant materials.

### Histomorphometric analysis

Digital images were acquired with the Panoramic MIDI Scanner. Specimens were magnified by 100 times to evaluate areas of newly formed bone. After creating a border along the defect and normal bone areas, the region containing newly formed bony tissues was converted into a percentage in which the defect area was regarded as 100.

### Measurement of interparticle distance of graft materials

Digital images were obtained with the Panoramic MIDI Scanner, and the closest interparticle distances for each type of graft material were measured in all samples.

### Reverse transcription polymerase chain reaction (RT-PCR)

Total RNA was extracted from each frozen tissue using the RNeasy Mini Kit (QIAGEN, Hilden, Germany), and the extracted RNA was put into a Transcriptor First Strand cDNA Synthesis Kit (Roche diagnostic, Seoul, Korea), which synthesizes 20 μL of complementary DNA (cDNA) from 250 ng of RNA. A total volume of 20 μL, which included 2 μL of cDNA and 1 μL of 10 μM F/R primer, was added to Maxime PCR PreMix (i-StarTaq, Intronbiotechnology, Seongnam, Korea). As for PCR conditions (Table 1), the gene expressions of osteonectin and osteopontin were analyzed, with GAPDH used as a housekeeping gene. Information about GAPDH and the primer sequences of osteonectin and osteopontin is shown in Tables 2 and 3.

**Table 1:** PCR sequence

Cycle	Temperature (°C)	Time
1	95	5 min
30	94	15 s
	60	15 s
	72	30s
1	72	10 min
1	4	∞

**Table 2:** Osteonectin and osteopontin primer sequence

Primer	Sequence
Osteonectin F	CTCCACCTGGACTACATCG
Osteonectin R	GCTGGCCAAACTGCCAGTG
Osteopontin F	GCTCAGCACCTGAATGTACC
Osteopontin R	CTTCGGCTCGATGGCTAGC

**Table 3:** GAPDH (NM_001082253.1) primer

Primer	Length	Sequence
GAPDH F	18	CACCCAGAAGACCGTGGA
GAPDH R	18	GTTCAGCTCGGGGATGAC

### Statistical analysis

Statistical analysis was performed using SPSS version 18 (SPSS, Chicago, IL, USA). The repeated measure ANOVA was conducted to compare new bone formation in each group on histomorphometric analysis. The statistical significance level was *p* < 0.05.

## Results

### Histology

**Fig. 3:**
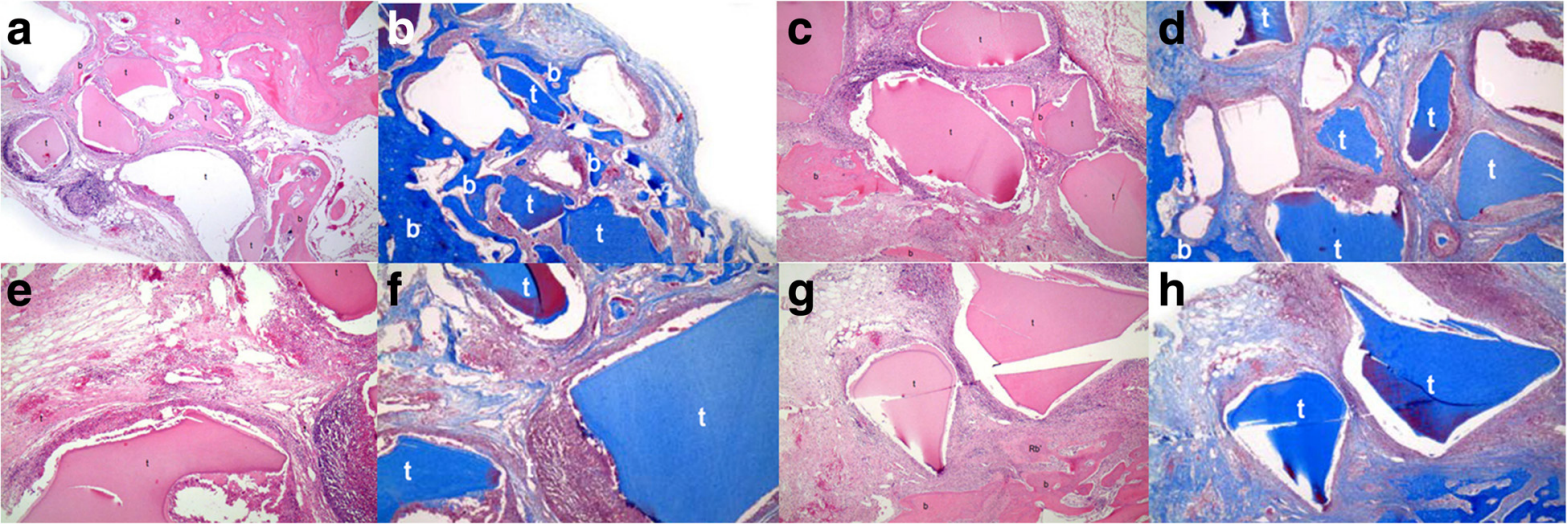
The histological examination. **a** Histologic finding of group 1 after 2 weeks (H-E stain, ×40): *t* DDM, *b* bone of skull, *rb* regenerative bony tissue, *Rb* regenerative bony tissue from the skull bone. **b** Histologic finding of group 1 after 2 weeks (M-T stain, ×40). **c** Histologic finding of group 2 after 2 weeks (H-E stain, ×40). **d** Histologic finding of group 2 after 2 weeks (M-T stain, ×40). **e** Histologic finding of group 3 after 2 weeks (H-E stain, X40). **f** Histologic finding of group 3 after 2 weeks (M-T stain, ×40). **g** Histologic finding of group 4 after 2 weeks (H-E stain, ×40). **h** Histologic finding of group 4 after 2 weeks (M-T stain, ×40)

At first, we examined the representative histology of each group of specimens. At 2 weeks, group 1 exhibited multinucleated giant cells, infiltrated lymphocytes, eosinocytes, macrophages, and a slight proliferation of connective tissue along the polygonal bone graft materials. Sites where existing bone tissue and bone graft materials were bound together could be seen in some areas. However, new bone formation was not observed (Fig. 3a, b). Group 2 showed an inflammatory response, with eosinocytes, lymphocytes, and macrophages surrounding bone graft materials, as well as proliferating connective tissue, and some areas contained existing bone tissue bound to bone graft materials (Fig. 3c, d). Group 3 showed a severe inflammatory response, the area being infiltrated with macrophages, lymphocytes, and eosinocytes, and bleeding was observed surrounding the bone graft materials. In some cases, multinucleated giant cells were noted as were the proliferation of connective tissue and inflammation between the materials (Fig. 3e, f). Similarly, group 4 showed an inflammatory response, with eosinocytes, lymphocytes, and macrophages surrounding the bone graft materials, and there was a noticeable proliferation of connective tissue. Although the new bone had formed from the existing bone, it had not formed between the materials (Fig. 3g, h).

**Fig. 4:**
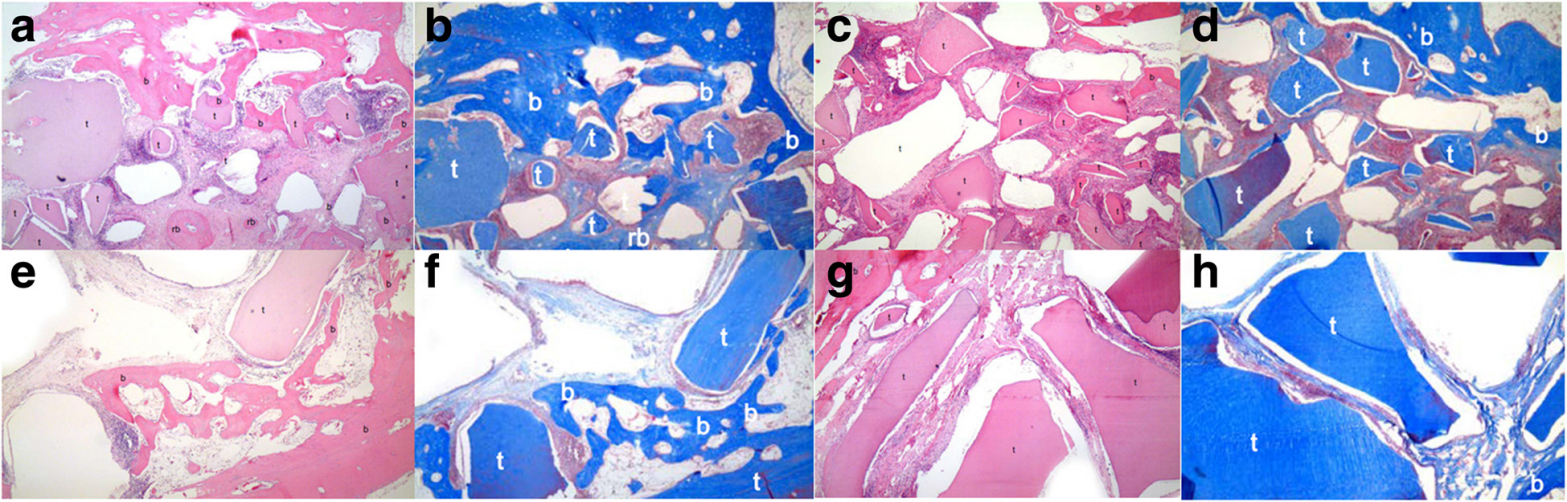
The histological examination. **a** Histologic finding of group 1 after 4 weeks (H-E stain, ×40). **b** Histologic finding of group 1 after 4 weeks (M-T stain, ×40). **c** Histologic finding of group 2 after 4 weeks (H-E stain, ×40). **d** Histologic finding of group 2 after 4 weeks (M-T stain, ×40). **e** Histologic finding of group 3 after 4 weeks (H-E stain, ×40). **f** Histologic finding of group 3 after 4 weeks (M-T stain, ×40). **g** Histologic finding of group 4 after 4 weeks (H-E stain, ×40). **h** Histologic finding of group 4 after 4 weeks (M-T stain, ×40)

At 4 weeks, lymphocytes and a few macrophages had infiltrated the materials in group 1. The proliferating dense connective tissue showed a noticeable increase in fibroblasts, and in some areas, the fibroblasts had been transformed into osteoblasts and formed osteoid for new bone growth. Binding between existing bone tissue and bone graft materials was easily detected, and the new bone had appeared between the bone graft materials (Fig. 4a, b). In group 2, lymphocytes, macrophages, and a few multinucleated giant cells had infiltrated the materials, and there was a noticeable increase in connective tissue, which was dense in some parts, with a small amount of bone tissue between the connective tissue and the materials. However, there was no binding between the new bone generated from existing bone and the materials (Fig. 4c, d). Group 3 showed lymphocytes, macrophages, and a few multinucleated giant cells infiltrated among the materials. Fibrous tissue had increased slightly, but there was no binding between the new bone generated from the existing bone and the materials (Fig. 4e, f). In group 4, lymphocytes and macrophages had infiltrated the materials, and there was a slight increase in fibrous tissue, with no new bone formation between the materials; however, the bone tissue and the materials showed some binding (Fig. 4g, h).

**Fig. 5:**
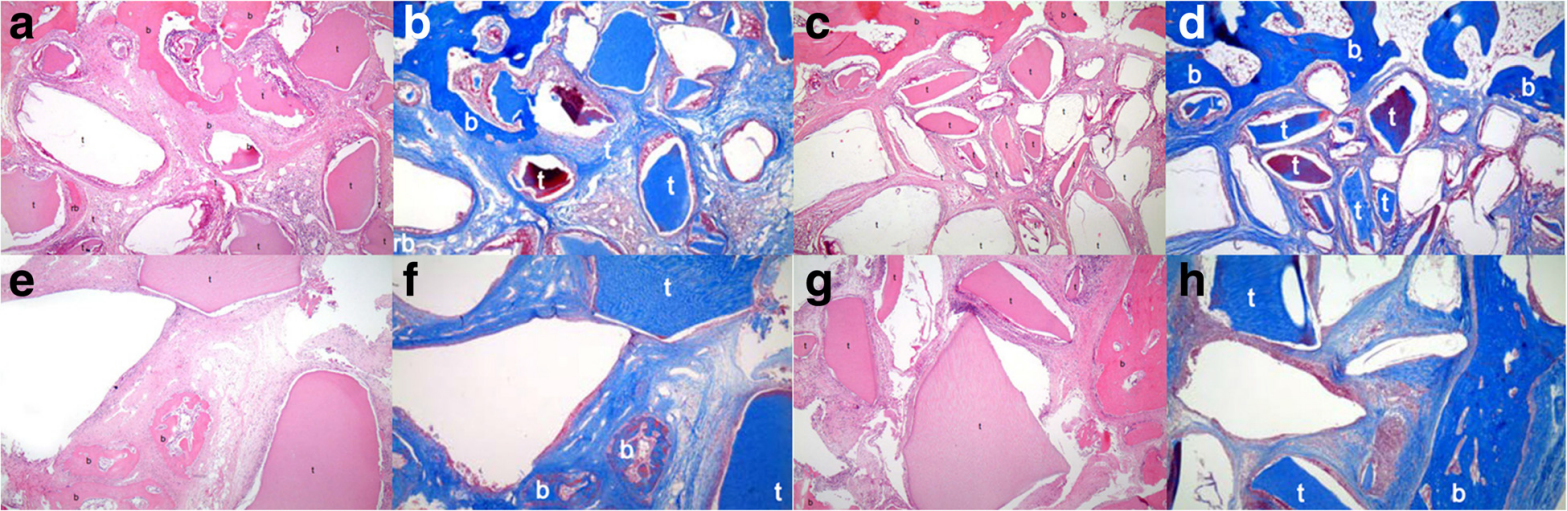
The histological examination. **a** Histologic finding of group 1 after 8 weeks (H-E stain, ×40). **b** Histologic finding of group 1 after 8 weeks (M-T stain, ×40). **c** Histologic finding of group 2 after 8 weeks (H-E stain, ×40). **d** Histologic finding of group 2 after 8 weeks (M-T stain, ×40). **e** Histologic finding of group 3 after 8 weeks (H-E stain, ×40). **f** Histologic finding of group 3 after 8 weeks (M-T stain, ×40). **g** Histologic finding of group 4 after 8 weeks (H-E stain, ×40). **h** Histologic finding of group 4 after 8 weeks (M-T stain, ×40)

At 8 weeks, group 1 showed infiltration of the materials by lymphocytes, macrophages, a few plasma cells and eosinocytes, and some multinucleated giant cells. Dense connective tissue had formed between the bone graft materials, and close to these materials, where fibrin was dense, osteoblasts, which had become differentiated from fibroblasts and had formed osteoid and then new bones (Fig. 5a, b). Group 2 showed less inflammation at 8 weeks than at 4 weeks, including macrophages, multinucleated giant cells, and lymphocytes, and denser connective tissue was found between the materials. Still, there was no binding between the new bone generated from the existing bone and the bone graft materials (Fig. 5c, d). Group 3 showed a slight inflammatory response, with macrophages, lymphocytes, and eosinocytes visible among the materials, in addition to noticeable dense connective tissue. The new bone was found between the materials, and activated osteoblasts could be seen within the bone tissue; however, the bone tissue was presumed to have formed from the existing bone (Fig. 5e, f). Group 4 showed a slight inflammatory response, with macrophages, lymphocytes, and eosinocytes present among the materials, and there was a more noticeable increase in connective tissue; however, new bone formation between the materials was not observed (Fig. 5g, h).

### Histomorphometric analysis

The percentages of the new bone that had formed in the rabbit skull defects at 2, 4, and 8 weeks, respectively, were 29.53 ± 7.65 %, 36.31 ± 9.80 %, and 52.27 ± 4.44 % in group 1; 27.02 ± 15.46 %, 34.54 ± 14.51 %, and 49.02 ± 6.75 % in group 2; 23.24 ± 8.95 %, 35.58 ± 5.59 %, and 46.99 ± 8.15 % in group 3; and 23.98 ± 5.56 %, 34.43 ± 12.87 %, and 44.96 ± 6.41 % in group 4. Although the results of these experiments at 2, 4, and 8 weeks all showed higher percentages of new bone formation in group 1 (0.1 mL of 0.25- to 1.0-mm particles), the differences compared with the other three groups were not statistically significant (*p* > 0.05) (Table 4).

**Table 4:** New bone formation (%)

	2 weeks	4 weeks	8 weeks
Group 1	29.53 ± 7.65	36.31 ± 9.80	52.27 ± 4.44
Group 2	27.02 ± 15.46	34.54 ± 14.51	49.02 ± 6.75
Group 3	23.24 ± 8.95	35.58 ± 5.59	46.99 ± 8.15
Group 4	23.98 ± 5.65	34.43 ± 12.87	44.96 ± 6.41

### Measuring the distance between materials

These distances were 209.06 ± 161.25 μm for group 1, 126.84 ± 79.19 μm for group 2, 880.42 ± 579.63 μm for group 3, and 299.42 ± 153.52 μm for group 4 (Table 5).

**Table 5:** Particle distance (μm)

	Group 1	Group 2	Group 3	Group 4
Distance	209.06 ± 161.25	126.84 ± 79.19	880.42 ± 579.63	299.42 ± 153.52

### RT-PCR analysis

**Fig. 6:**
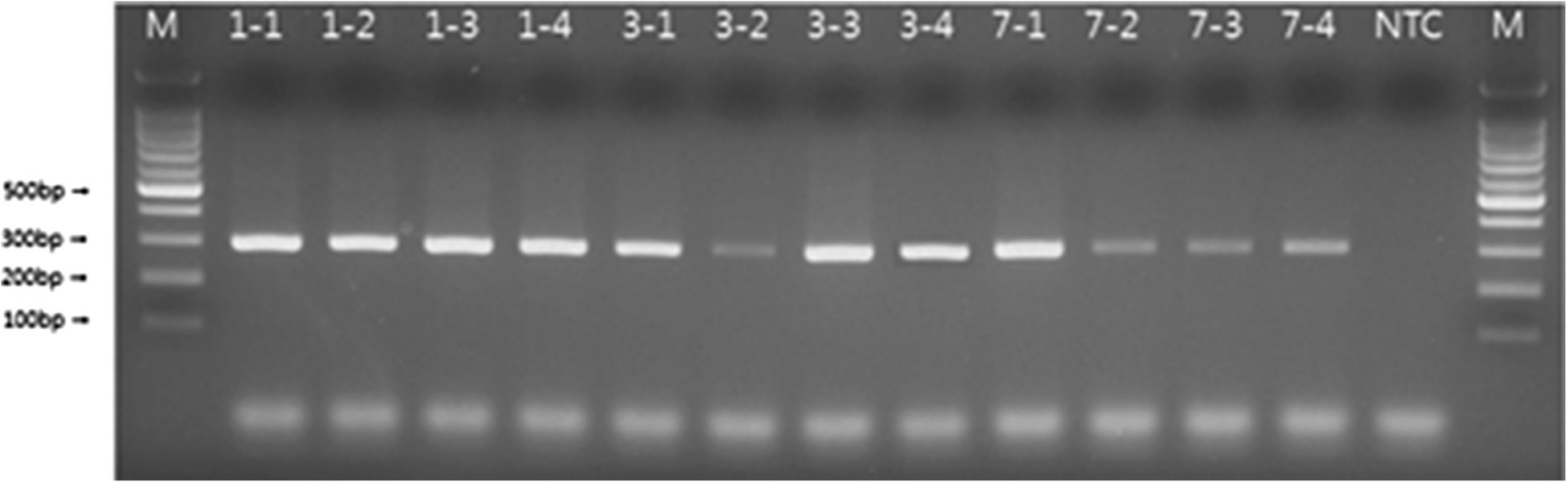
RT-PCR (osteonectin). *M* size marker, *NTC* no template control

**Fig. 7:**
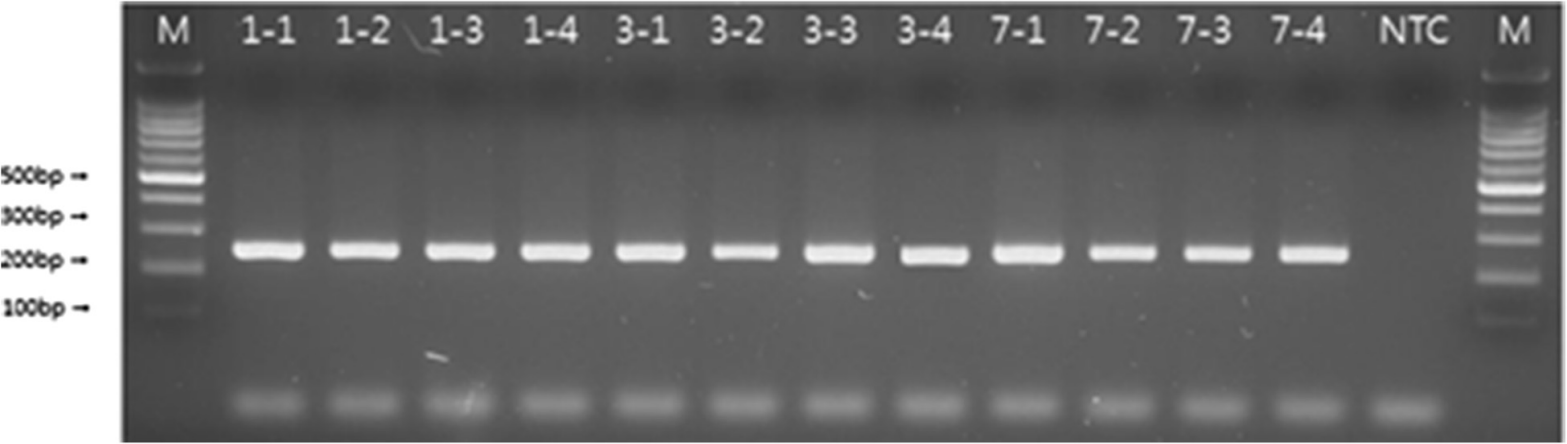
RT-PCR (osteopontin)

At 2 weeks, the expression of osteonectin was similar in all four groups of specimens. At 4 weeks, group 3 showed slightly greater osteonectin expression, and at 8 weeks, group 1 showed greater expression (Fig. 6). On the analysis of osteopontin expression, there was little difference between each of the three periods, but after 2 weeks, expression of osteopontin was similar to that of osteonectin. Osteopontin expression was increased in group 3 at 4 weeks and in group 1 at 8 weeks (Fig. 7).

## Discussion

Bone grafting is apparently used often in the oral and maxillofacial area to enhance aesthetic and functional recovery, stabilization, and healing. The basic objective of such grafting is to maintain the biomechanical role of bone by restoring its morphology and physiological function.

Bone graft materials are known to form bones in three ways. First, in osteogenesis, viable osteoblasts and pre-osteoblasts in the materials induce an ossification reaction around the graft sites to form new bone tissue. The amount of newly formed bones is proportionate to that of living graft cells. Osteogenesis is usually seen in cancellous bones and after bone marrow transplantation. A second process for creating new bone is osteoinduction. Chemotactic materials such as BMP induce the growth of undifferentiated mesenchymal cells around the graft site, and these cells are converted into chondroblasts and osteoblasts to form new bones. Third, through a process of osteoconduction, the bone graft materials function simply as a scaffold that enables vascularization of the site, and the differentiated mesenchymal cells around the site provide the chondroblasts and osteoblasts needed to form the new bone using grafting materials. New bones are formed by means of creeping substitution, which refers to the resorption of the graft materials along with the deposition of the new bone. The presence of the bone around the graft site and of differentiated mesenchymal cells is essential for osteoconduction [29–31].

The key ingredient in bone grafting subsequent to implant surgery is bone remodeling in order to withstand the occlusal load. Bone remodeling continuously proceeds through the delicate regulation of two contrasting procedures: bone resorption by osteoclasts and bone formation by osteoblasts. Bone tissue formed with the help of bone graft materials is dynamic, having the enhanced regenerative ability to continue to undergo remodeling according to the internal or external conditions.

Many studies have altered bone graft materials in attempts to improve the reactions of the bone. Among them have been reports indicating that bone graft materials with different particle sizes exhibit different bone-healing abilities [16–19]. Although still controversial, these results suggest that the smaller the particle size of the material, the greater the formation of bone. The reasons for this are said to be that smaller particles increase the available surface area, and more growth factors of various kinds are secreted to facilitate the formation of new blood vessels and accelerate the differentiation of mesenchymal cells into osteoblasts, thus assisting the process of bone formation.

In addition, it has been reported that the spaces between particles should be more than 100 μm in order to achieve proper vascularization and bone formation [32, 33]. According to some researchers, spaces of 300 to 500 μm between particles led to greater formation of bone as compared with spaces of 50 to 100 μm [34, 35]. In this context, in bone formation experiments using hydroxyapatite, an interconnected porous structure between particles was found to be a significant factor in osteoconduction and synostosis [36, 37]. Thus, both a porous structure and particle size are critical factors in that they enhance bone-forming ability and affect the period of resorption.

Previous reports indicated an association between the particle size of bone grafting materials and the success of bone formation, and particles that are 0.25 to 1.0 mm and 1 to 2 mm in size are available on the domestic market. The DDM used in our study was also manufactured in these sizes, and both the porous structure and space between the bone grafting materials were considered to affect the ability of bone formation. Therefore, in this study, we tested materials of different densities to evaluate the effects of different distances between the bone graft materials. As a result, we found that particles 0.25 to 1.0 mm in size and spaces of 200 μm between the materials activated bone formation the most.

Owing to the considerable inflammatory response and lack of bone formation observed in the early stages after grafting, we believe that the use of human DDM in the animal model should be regarded not as autologous but as heterologous tissue grafting.

As analyzed on RT-PCR, osteonectin has been found in various kinds of cells, but when detected in adult cells, it has appeared only in calcified tissues. Previously, osteonectin was believed to have an affinity to bind to apatite and collagen type 1 that temporarily accelerates skeletal calcification. However, SPARC, or osteonectin/BM-40 (secreted protein acidic and rich in cysteine), is from the basement membrane and contains the same amino acid sequences as osteonectin, so the role of osteonectin in calcification is now regarded negatively. Osteopontin is a phosphoprotein that constitutes the framework. It contains sialic acid and is referred as sialoprotein-1 (BSP-1). Its high-binding affinity with calcium offers an explanation for the formation of calcified tissues or modeling. Protein from osteopontin has several calcium binding sites and such binding converts it to a three-dimensional structure that is related to certain functions. In the present study, the concentrations of osteonectin and osteopontin were elevated until the eight week, indicating the active formation of bone.

## Conclusions

In conclusion, we found that small particles of bone grafting material measuring 0.25 to 1.0 mm with 200 μm of space between the materials were effective in promoting osteogenesis. In our study, the space between the bone graft materials was considered to be two-dimensional and was measured accordingly. Thus, future studies should consider a three-dimensional evaluation of the space as well as other options for determining the amount of materials needed for successful bone formation.
